# MicroRNA-497 increases apoptosis in *MYCN* amplified neuroblastoma cells by targeting the key cell cycle regulator *WEE1*

**DOI:** 10.1186/1476-4598-12-23

**Published:** 2013-03-26

**Authors:** Laura Creevey, Jacqueline Ryan, Harry Harvey, Isabella M Bray, Maria Meehan, Adnan R Khan, Raymond L Stallings

**Affiliations:** 1 Department of Molecular and Cellular Therapeutics, Royal College of Surgeons in Ireland, York House, York Street, Dublin 2, Ireland; 2 National Children’s Research Centre, Our Lady’s Children’s Hospital, Crumlin, Dublin 12, Ireland; 3 Trinity Biomedical Sciences Institute, School of Medicine, Trinity College, Dublin, Ireland

**Keywords:** miR-497, Neuroblastoma, *WEE1*, Tumor suppressor, Cisplatin

## Abstract

**Background:**

Neuroblastoma is responsible for 15% of all childhood cancer deaths. Despite advances in treatment and disease management, the overall 5-year survival rates remain poor in high-risk disease (25-40%). MiR-497 was previously identified by our laboratory as a member of a miRNA expression signature, predictive of neuroblastoma patient survival and has been reported as a tumor suppressor in a variety of other cancers. *WEE1*, a tyrosine kinase regulator of the cell cycle and predicted target of miR-497, has emerged as an oncogene in several cancer types and therefore represents an attractive potential target for novel therapy approaches in high-risk neuroblastoma. Our aim was to investigate the potential tumor suppressive role of miR-497 in high-risk neuroblastoma.

**Methods:**

Expression levels of miR-497 and *WEE1* in tissues and cells were determined using RT-PCR. The effect of miR-497 and siWEE1 on cell viability was evaluated using MTS assays, apoptosis levels were determined using FACS analysis of Annexin V/PI stained cells, and target protein expression was determined using western blot. Luciferase reporter plasmids were constructed to confirm direct targeting. Results were reported as mean±S.E.M and differences were tested for significance using 2-tailed Students t-test.

**Results:**

We determined that miR-497 expression was significantly lower in high-risk *MYCN* amplified (MNA) tumors and that low miR-497 expression was associated with worse EFS and OS in our cohort. Over-expression of miR-497 reduced cell viability and increased apoptosis in MNA cells. We identified *WEE1* as a novel target for miR-497 in neuroblastoma. Furthermore, our analysis showed that high *WEE1* levels are significantly associated with poor EFS and OS in neuroblastoma and that siRNA knockdown of *WEE1* in MNA cell lines results in significant levels of apoptosis, supporting an oncogenic role of *WEE1* in neuroblastoma. Cisplatin (CDDP) treatment of both miR-497 over-expressing cells and *WEE1* inhibited cells, resulted in a significant increase in apoptosis in MNA cells, describing a synergistic effect and therefore a potential therapeutic for high-risk neuroblastoma.

**Conclusion:**

Our study’s results are consistent with miR-497 being a candidate tumor suppressor in neuroblastoma, through the direct targeting of *WEE1*. These findings re-enforce the proposal of *WEE1* as a therapeutic target in neuroblastoma.

## Introduction

Neuroblastoma, a paediatric cancer that originates from precursor cells of the sympathetic nervous system, is responsible for 15% of all childhood cancer deaths [1]. Tumors display a high level of heterogeneity, with clinical outcome ranging from spontaneous regression without treatment, to rapid disease progression and mortality [2,3]. Patients are risk stratified according to the identification of several prognostic factors at diagnosis including; level of disease dissemination (defined by the International Neuroblastoma Staging System (INSS)), age, histology, and presence of high-risk genetic features such as amplification of the *MYCN* proto-oncogene and chromosomal gains (17q) and deletions (11q or 1p) [1,4]. Despite advances in treatment and disease management, the overall 5-year survival rates remain poor in high-risk disease (25-40%). Further elucidation of the underlying mechanisms of neuroblastoma disease, and recent advances in understanding the molecular basis of high-risk neuroblastoma may contribute to a greater understanding of response to therapy and outcome, potentially leading to the identification of suitable therapeutic targets that may respond to novel agents [5,6].

MicroRNAs (miRNAs) are a class of short non-coding RNAs that have emerged as significant epigenetic regulators of cellular functions, predominantly through silencing of their target genes via direct complementary mRNA 3′UTR base pairing. Dysregulation of miRNAs has been reported in numerous cancers where individual miRNA behave in an oncogenic or tumor suppressor manner [7,8]. To date, several profiling studies have identified miRNAs that are associated with clinical outcome in neuroblastoma [9-13] and specific miRNAs have been identified that regulate key processes such as apoptosis, differentiation, cell proliferation and cell invasiveness in neuroblastoma [14-17].

MiR-497 was previously identified by our laboratory as a member of a miRNA expression signature that is predictive of neuroblastoma patient survival [9], and has also been reported to play a tumor suppressor role in a variety of other cancers [18-20]. Down-regulation of miR-497 has been reported in both multidrug resistant lung and gastric cancer cell lines, compared to non-resistant cell lines [21]. Recently, *BCL2* (a known anti-apoptotic protein determined to be involved with neuroblastoma drug resistance) has been demonstrated as a direct target of miR-497 in neuroblastoma cells [22], further highlighting an important tumor suppressor role of this miRNA in this cancer.

*WEE1*, a tyrosine kinase regulator of the cell cycle, is over-expressed in several cancer types, including hepatocellular carcinoma and breast cancer and is also associated with poor disease free survival in malignant melanoma [23-25]. *WEE1* expression has been demonstrated to prevent ovarian cancer cells from undergoing apoptosis in response to DNA damage [26]. *WEE1* inhibition, in breast cancer, results in a significant decrease in cell proliferation and increased apoptotic levels. This effect is mirrored by inhibition of *WEE1* in cells exposed to DNA damaging agents in glioblastoma [27,28].

Here we report that low miR-497 expression levels are associated with event free survival (EFS) and overall survival (OS) in our neuroblastoma cohort and describe a significant difference in miR-497 expression between *MYCN*-amplified (MNA) versus non-*MYCN*-amplified (non-MNA) tumors. We demonstrate that miR-497 over-expression results in significantly decreased cell viability through increased apoptotic rates in MNA neuroblastoma cells, in part, through the direct targeting of the 3′UTR of *WEE1*. Furthermore, we observe that higher than median *WEE1* levels are significantly associated with poor EFS and OS in neuroblastoma and that siRNA knockdown of *WEE1* in MNA neuroblastoma cell lines results in significant and profound levels of apoptosis, supporting an oncogenic role of *WEE1* in neuroblastoma. Treatment of either miR-497 over-expressing cells or *WEE1* inhibited cells with CDDP resulted in a significant increase in apoptotic rates in MNA neuroblastoma cells. The synergistic enhancement of CDDP induced apoptosis through miRNA or siRNA mediated *WEE1* inhibition indicates a potential therapeutic strategy for high risk neuroblastoma.

## Results

### MiR-497 expression is significantly associated with event free and overall survival in neuroblastoma

**Figure 1:**
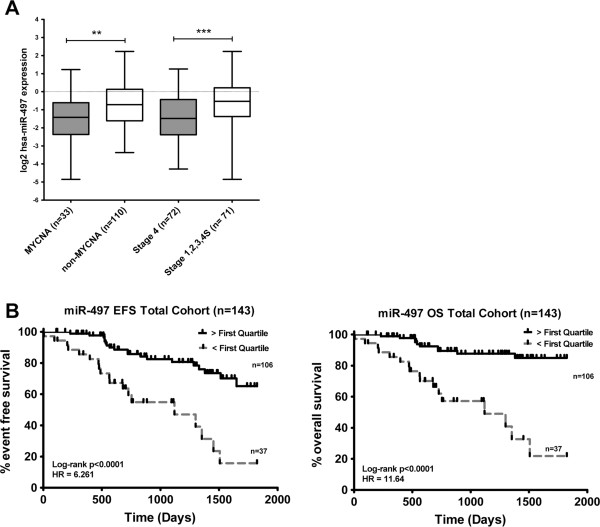
**miR-497 expression is significantly associated with risk and survival in a neuroblastoma cohort (n=143). (A)** Box and whiskers plots represent the expression of miR-497 in a cohort of 143 primary neuroblastoma tumors. Samples are grouped according to known risk factors and expression compared in each of; *MYCN* amplified versus Non *MYCN* amplified or Stage 4 versus INSS Stage 1,2,3,4S. Statistical differences in median expression were analysed using two sided Mann–Whitney *U* tests. Dark shading indicates known higher risk factor. HR= Hazard Ratio. **(B)** Kaplan-Meier plots for event free survival (EFS) and overall survival (OS) in 143 neuroblastoma patients. *P* values were obtained using log-rank test.

Analysis of miR-497 expression levels in 143 primary diagnostic neuroblastoma samples (Additional file 1: Table S1) revealed significantly lower expression of miR-497 (based on median expression) in patients with known higher risk prognostic factors including MYCN amplification (MNA) and INSS Stage 4 disease (Figure 1A). Although miR-497 was found not to be independent of other known risk factors (Additional file 2: Table S2), higher expression (> first quartile) of miR-497 was significantly associated with both improved event free survival (EFS 5 year 65% vs 16%) and overall survival (OS 5 year 84% vs 21%), indicating a potential tumor suppressor role in neuroblastoma (Figure 1B).

### Ectopic expression of miR-497 decreases cell viability and increases apoptotic rates in neuroblastoma cells *in vitro*

**Figure 2:**
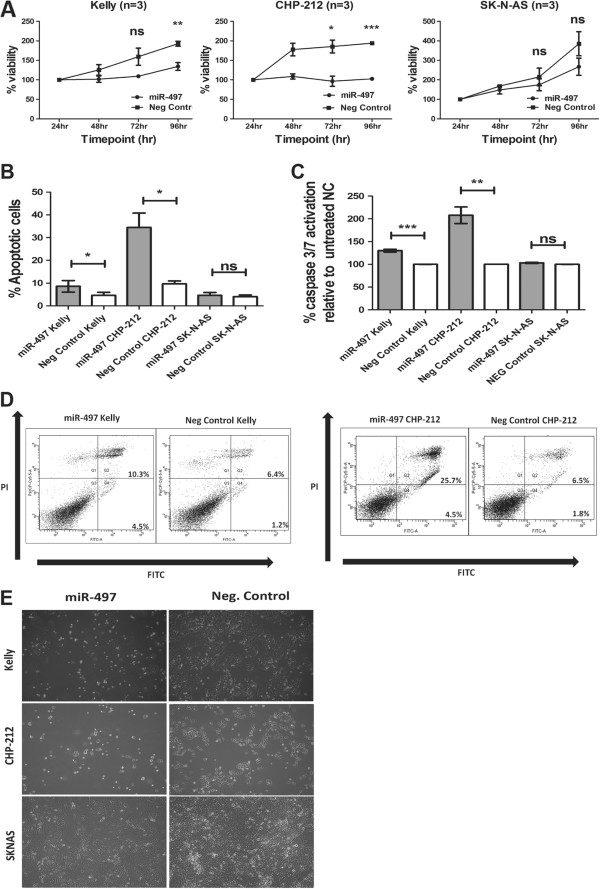
**Ectopic expression of miR-497 decreases cell viability and increases apoptotic rates *in vitro. *(A)** Neuroblastoma *MYCN*-amplified cell lines Kelly (n=3), CHP-212 (n=3) and non-*MYCN*-amplified SK-N-AS (n=3) were transfected with miR-497 mimics/scrambled negative control (Neg Control) oligonucleotides. Viability of cells was measured by MTS assay at 24 hr, 48 hr, 72 hr and 96 hr post transfection. **(B)** Mean percentage of annexin V^+^ Kelly, CHP-212 and SK-N-AS cells, from at least three independent experiments at 96 hr post transfection with miR-497 mimic/Scrambled NC. **(C)** Caspase 3/7 activation was measured at 72 h post transfection (n=3, each × 5 technical replicates) in Kelly, CHP-212 and SK-N-AS cell lines, (**D**) Representative Annexin V Scatter plots for neuroblastoma *MYCN*-amplified Kelly and CHP-212 cells following transfection with miR-497 mimics/scrambled negative control (Neg Control) oligonucleotides. (**E**) Images of miR-497 over-expressing cells vs scrambled negative controls (Neg Control) in Kelly, CHP-212 and SK-N-AS.

To further investigate a potential tumor suppressor role of miR-497 in neuroblastoma, we observed the effects of miR-497 over-expression on neuroblastoma cell viability, by transiently transfecting mature miR-497 mimics into MNA Kelly and CHP-212 cell lines and non-MNA SK-N-AS cell line. MiR-497 expression was significantly up-regulated following transfection in all cell lines (Additional file 3: Figure S1). Ectopic expression of miR-497 resulted in significantly decreased cell viability in the MNA Kelly and CHP-212 cell lines at 96 hr relative to negative controls, while lowered cell viability observed in SK-N-AS was not statistically significant (Figure 2A).

**Figure 3:**
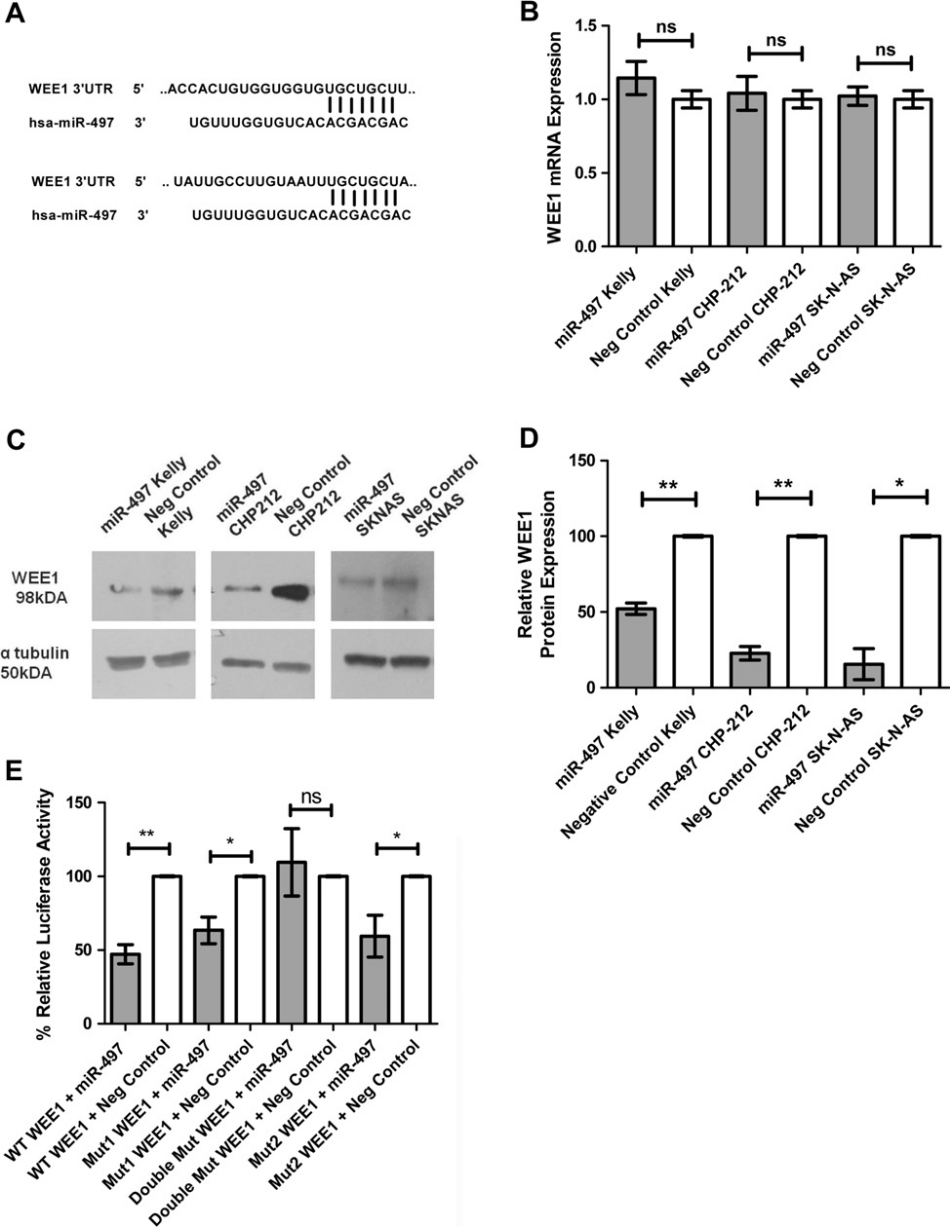
**MiR-497 targets the 3′UTR of *WEE1 *in neuroblastoma cells. (A) ** Two conserved 7-mer seed matches with hsa-miR-497 in *WEE1* 3′ UTR. **(B)***WEE1* mRNA expression levels 24 hours after transfection with miR-497 compared to negative controls in neuroblastoma *MYCN*-amplified cell lines Kelly (n=3), CHP-212 (n=3) and non-*MYCN*-amplified SK-N-AS (n=3). **(C)** WEE1 protein levels, following transfection with miR-497 mimics or scrambled negative control, determined by western blot of protein extraction at 96 hrs. **(D)**Densitometry analysis of WEE1 protein levels following miR-497 over-expression in Kelly, CHP-212 and SK-N-AS cells. **(E)** Kelly cells were co-transfected with miR-497 mimics or Scrambled negative control and either wild-type (WT) or mutated (mut) *WEE1* 3′UTR reporter constructs. Luciferase activity was determined 48 hrs post transfection.

Consistent with these results, a significant increase in apoptosis activity was observed in MNA Kelly and CHP-212 cells following miR-497 transfection, as determined by Annexin V/PI staining and FACs analysis at 96 hr following miR-497 transfection (Figure 2B). No change in the rate of apoptotic cell death was detected for SK-N-AS (Figure 2B). Representative scatter plots for Kelly and CHP-212 are illustrated (Figure 2D). A significant increase in caspase 3/7 activation was observed in MNA Kelly and CHP-212 cells when compared to negative controls. Consistent with both cell viability and Annexin V assays, no change in caspase activation was observed in non-MNA SK-N-AS cells (Figure 2C). We also note that Kelly and CHP-212 cells became rounded and detached following miR-497 over-expression, consistent with apoptosis, whereas SK-N-AS cells remained attached to the dish (Figure 2E). We conclude that miR-497 decreases cell viability in MNA neuroblastoma Kelly and CHP-212 cells through an increase in apoptotic rates. Consistent with the above results, cell cycle analysis of MNA Kelly cells revealed a significant increase in cell number in the G_0_/G_1_ phase of the cell cycle following over-expression of miR-497 when compared to negative controls. A corresponding significant decrease in the number of cells in the G_2_/M of the cell cycle was observed, following over-expression of miR-497 when compared to negative controls. These results further support the apoptotic phenotype following miR-497 over-expression (Additional file 4: Figure S4A). Representative cell cycle analysis plots of MNA Kelly cells are illustrated (Additional file 4: Figure S4C). MiR-497 targets the 3′UTR of *WEE1* in neuroblastoma cells. Through *In silico* analysis (using the algorithms TargetScan, miRDB and miRanda), we examined all computationally predicted target genes of miR-497. *WEE1* was a computationally predicted target of interest given our observed phenotypic effect of decreased cell viability and increased apoptotic rates following miR-497 over-expression, and *WEE1*’s documented role as a negative regulator of CDC2 mediated apoptosis [29]. *WEE1* has two conserved 7-mer seed matches with miR-497 in its 3′UTR (Figure 3A).

Ectopic expression of miR-497 mimics in Kelly, CHP-212 and SK-N-AS cells resulted in knockdown of WEE1 protein but not mRNA, indicating that the miRNA had a potential inhibitory effect on translation (Figure 3B,C). Densitometry analysis shows significant decrease in WEE1 protein levels following miR-497 over-expression when compared to negative controls (Figure 3D). To determine if miR-497 directly targets the 3′ UTR of *WEE1,* luciferase reporter plasmids were constructed containing a 450 bp segment of the *WEE1* 3′ UTR with either the wild type or a mutated miR-497 seed site. As miR-497 has two potential binding sites in the 3′UTR of *WEE1*, mutant reporter constructs were made that had either single mutated sites or both sites mutated (Additional file 5: Figure S2). Co-transfection of the reporter construct containing the wild-type binding sequence with mature miR-497 mimics resulted in a statistically significant reduction in luciferase activity in Kelly cells (Figure 3E). Luciferase activity was also significantly reduced in both reporter constructs with only one of the two potential miR-497 binding sites mutated. This effect was abrogated by a double mutated target sequence, thereby confirming that *WEE1* is directly targeted by miR-497 (Figure 3E).

### siRNA mediated *WEE1* knockdown represents a potent mechanism of apoptosis induction in neuroblastoma cells *in vitro*

**Figure 4:**
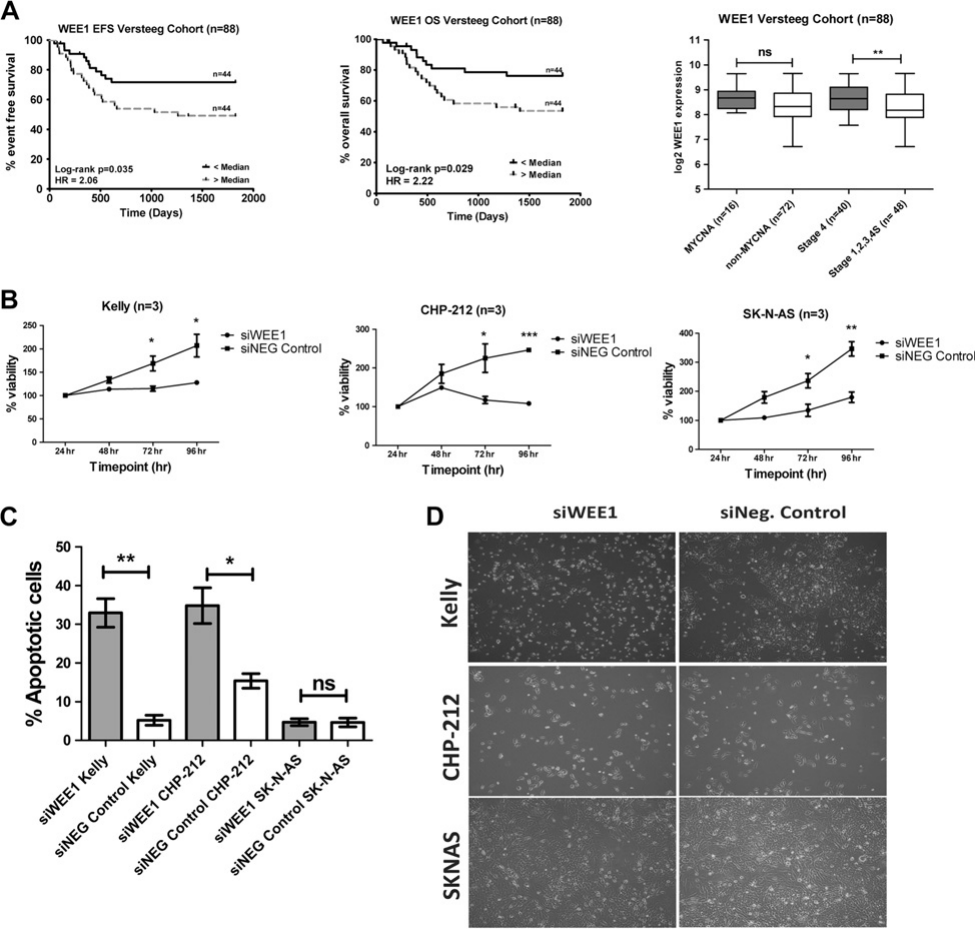
**siRNA mediated inhibition of *WEE1 *results in decreased cell viability and increased apoptosis *in vitro.*(A)** Kaplan-Meier plots for EFS and OS in 88 neuroblastoma patients (Amsterdam) based on median *WEE1* mRNA expression. *P* values were obtained using log-rank test. Box and whiskers plots represent the expression of *WEE1* in a cohort of 88 primary neuroblastoma tumors. Samples are grouped according to known risk factors and expression compared in each of; *MYCN* amplified versus Non *MYCN* amplified and INSS Stage 4 versus INSS Stage 1,2,3,4S. Statistical differences in median expression were analysed using two sided Mann–Whitney *U* tests. Dark shading indicates known higher risk factor. HR= Hazard Ratio. **(B)** Neuroblastoma *MYCN*-amplified cell lines Kelly (n=3), CHP-212 (n=3) and non-*MYCN*-amplified SK-N-AS (n=3) were transfected with siWEE1 /siNegative control (siNeg Control). Viability of cells was measured by MTS assay at 24 hr, 48 hr, 72 hr and 96 hr post transfection. **(C)** Mean percentage of annexin V^+^ Kelly, CHP-212 and SK-N-AS cells, from at least three independent experiments at 96 hr post transfection with siWEE1 /siNegative control (siNeg Control). **(D)** Images of siWEE1 inhibited cells versus siNegative controls (siNeg Control) in Kelly, CHP-212 and SK-N-AS.

Analysis of *WEE1* expression levels in 88 primary diagnostic neuroblastoma samples revealed a significant association of high *WEE1* expression with poor EFS and OS. Further analysis of this data set revealed no significant difference in the median expression of *WEE1* in tumors with/without MNA but a significant difference for *WEE1* median expression levels between tumors from INSS Stage 4 versus Stage 1,2,3 and 4 s disease (Figure 4A).

To test the hypothesis that miR-497 targeting of *WEE1* might contribute, in part, to the biological effects of miR-497, we performed siRNA mediated inhibition of *WEE1*. *WEE1* was significantly reduced at both mRNA and protein levels following siWEE1 transfection of Kelly, CHP212 and SK-N-AS cells (Additional file 6: Figure S3A and B). siWEE1 resulted in significantly decreased cell viability in Kelly, CHP-212 and SK-N-AS cell lines at both 72 hr and 96 hr time points relative to negative controls (Figure 4B). Given that siRNA mediated inhibition of *WEE1,* a now validated target of miR-497, had a similar phenotypic effect on our neuroblastoma cell lines, Annexin V/PI assays were performed using siWEE1.

Following *WEE1* inhibition, significantly increased levels of apoptosis (Annexin V^+^) were observed relative to negative controls at 96 hr for both Kelly and CHP-212. However, in SK-N-AS cells, there was not a significant increase in apoptotic cell numbers (Figure 4C). siRNA mediated inhibition of *WEE1* in Kelly and CHP-212 cells resulted in the cells becoming rounded and detached, similar to the morphological changes observed following miR-497 over-expression. SK-N-AS cells, however, retained their morphology and remained attached to the dish, consistent with results produced by miR-497 ectopic over-expression. Taken together, our results indicate that *WEE1* knockdown mediated by either miR-497 or siRNA in SK-N-AS results in a reduction in cell proliferation without causing increased apoptosis (Figure 4D). Consistent with the above results, cell cycle analysis of MNA Kelly cells revealed a significant increase in cell number in the G_0_/G_1_ phase of the cell cycle following *WEE1* inhibition when compared to negative controls. A corresponding significant decrease in the number of cells in the G_2_/M of the cell cycle following *WEE1* inhibition was observed, when compared to negative controls. These results further support the apoptotic phenotype following *WEE1* inhibition (Additional file 4: Figure S4B). Representative cell cycle analysis plots of MNA Kelly cells are illustrated (Additional file 4: Figure S4D).

### CDDP treatment combined with ectopic expression of miR-497 or siRNA mediated knockdown of *WEE1* results in increased apoptotic rates in neuroblastoma cells *in vitro*

**Figure 5:**
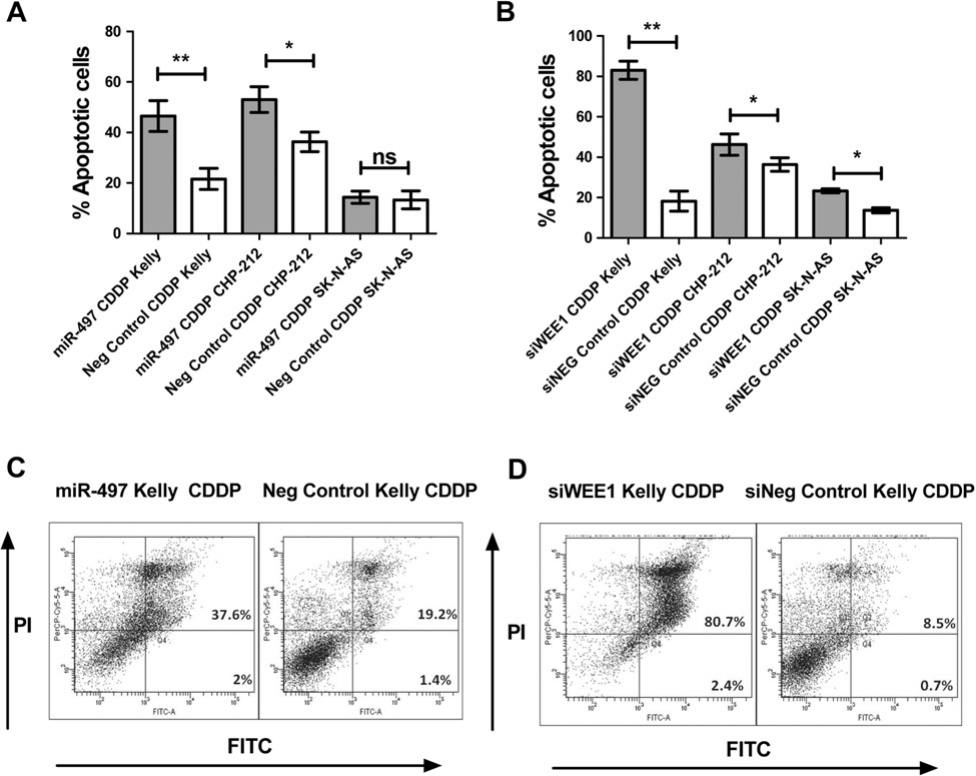
**Cells transfected with miR-497 mimics or siWEE1 followed by CDDP treatment. (A)** Neuroblastoma *MYCN*-amplified cell lines Kelly, CHP-212 and non-MNA SK-N-AS were transfected with miR-497 mimics/scrambled negative control (Neg Control) oligonucleotides. At 48 hr post transfection, cells were treated for 24 hr with 5 μg/ml concentration of CDDP. Media was replaced for a further 24 hrs. Mean percentage of annexin V^+^ Kelly, CHP-212 and SK-N-AS cells, from at least three independent experiments at 96 hr post transfection **(B)** Neuroblastoma *MYCN*-amplified cell lines Kelly, CHP-212 and non-MNA SK-N-AS were transfected with siWEE1 /siNegative control (siNeg Control). At 48 hrs post transfection, cells were treated for 24 hr with 5 μg/ml concentration of CDDP. Media was replaced for a further 24 hr.Mean percentage of annexin V^+^ Kelly, CHP-212 and SK-N-AS cells, from at least three independent experiments at 96 hr post transfection **(C)** Representative Annexin V Scatter plots for neuroblastoma *MYCN*-amplified Kelly cells following transfection with miR-497 mimics/scrambled negative control (Neg Control) oligonucleotides and 5 μg/ml CDDP treatment. **(D)** Representative Annexin V Scatter plots for neuroblastoma *MYCN*-amplified Kelly cells following transfection with siWEE1 /siNegative control (siNeg Control) and 5 μg/ml CDDP treatment.

In order to elucidate how miR-497 may contribute to improved patient survival, we assessed the effect of miR-497 over-expression and siRNA mediated knockdown of *WEE1* on apoptotic rates in Kelly, CHP-212 and SK-N-AS cell lines in response to CDDP. Following transfection with miR-497 mimics and treatment with 5ug/ml CDDP, a significant increase in apoptotic cells (Annexin V^+^) was detected in Kelly and CHP-212 (Figure 5A) relative to negative controls. These cell lines also displayed increased apoptosis in response to siRNA mediated *WEE1* inhibition and treatment with 5ug/ml CDDP (Figure 5B). SK-N-AS exhibited a significant increase in apoptosis in response to siRNA mediated *WEE1* inhibition and treatment with 5ug/ml CDDP, but not in response to miR-497 and CDDP (Figure 5A,B), compared to negative controls. Representative scatter plots for Kelly are illustrated (Figure 5C,D).

## Discussion

The dysregulation of miRNAs is a key mechanism involved in the pathogenesis of neuroblastoma, with several tumor suppressor miRNAs having been identified [11,14-17,30,31]. Here we determined that expression of miR-497, another potential tumor suppressor in neuroblastoma, was significantly lower in high-risk *MYCN* amplified tumors and that lower miR-497 expression was associated with worse EFS and OS in our patient cohort. Similarly, miR-497 expression has been associated with improved patient survival in other cancers, including breast and colorectal cancer (CRC), suggesting a wider tumor suppressor role for this miRNA [18,19]. Ectopic expression of miR-497 decreased breast cancer cell proliferation and increased apoptosis in colorectal cancer cell lines [18,19]. Similarly, we observed a significant increase in apoptosis in our MNA neuroblastoma cell lines following over-expression of miR-497 *in vitro*.

To date, the characterisation of miR-497 function has not been extensive, although several targets have been identified with a key role in cell cycle and survival pathways, including *BCL2*, *CCND2* and *IGF1-R*[18,21,22]*.* We have identified a novel target of miR-497, *WEE1* tyrosine kinase, an emerging novel therapy target and potent pro-survival protein in neuroblastoma. *WEE1*, an important regulator at the G2 checkpoint, normally negatively regulates entry into mitosis through phosphorylation of Tyr15 on CDC2 [29]. Several studies have identified that *WEE1* is essential for normal cell function and embryonic development [32,33]. *WEE1* has been associated with survival in several other cancer types including glioblastoma, malignant melanoma and breast cancer [25,27,28]. This is consistent with our findings as the analysis of *WEE1* expression levels in an independent cohort of 88 primary diagnostic neuroblastoma samples revealed a significant association of high *WEE1* expression with poor EFS and OS.

An interesting study on CD34^+^ umbilical cord blood cells by Lei *et al.,* focused on the anti-apoptotic role of *WEE1*. They showed that when these cells were treated with chemotherapeutic agents, including CDDP, the over-expression of *WEE1* supported cell viability and resulted in decreased apoptosis [34]. Subsequent studies have identified that knockdown of *WEE1*, including siRNA mediated knockdown or the use of the novel *WEE1* inhibitor MK-1775, successfully enhances the response to chemotherapy through abrogation of G_2_ arrest and increased apoptosis [28,35]. The therapeutic application of combined MK-1775 and chemotherapy has been confirmed in preclinical studies and results of clinical studies evaluating MK-1775 are awaited [36].

Russell et al., described how down-regulation of WEE1 protein in neuroblastoma MNA cell lines resulted in significantly increased apoptosis, making this an attractive potential target for novel therapy approaches in high-risk neuroblastoma [37]. We determined that miR-497 directly targets and inhibits *WEE1* protein expression in neuroblastoma cell lines, resulting in increased apoptosis. Russell et al., reported that sensitivity to a *WEE1* inhibitor (MK-1775) correlated with MYCN dosage [37], consistent with our findings that miR-497 inhibition of *WEE1* produced a more significant increase in apoptosis in MNA neuroblastoma cell lines compared to the non-MNA SK-N-AS cell line. Even siRNA mediated *WEE1* inhibition did not result in an apoptotic increase for SK-N-AS, although a significant reduction in cell growth occurred. However, following siRNA *WEE1* knockdown and treatment with CDDP, a significant increase in apoptotic levels was recorded in both MNA and non-MNA cell lines. The different phenotypic response observed between MNA and non-MNA cell lines following siRNA mediated inhibition of *WEE1* and ectopic expression of miR-497, when combined with CDDP, may be reasonably explained. siWEE1 is specifically designed and has been optimised for maximal knock-down of WEE1, whereas, miR-497, although directly targeting *WEE1*, may act in concert with other regulatory factors. siRNA mediated knockdown of WEE1 is more potent than the knockdown of WEE1 observed following miR-497 over-expression (Figure 3C, 3D and Additional file 6: Figure S3B, S3Cf). Given the significantly increased level of WEE1 protein knockdown following transfection with siWEE1 when compared to the level of WEE1 protein knockdown following miR-497 over-expression, this may explain the significant increase in apoptotic levels in SK-N-AS when combined with CDDP treatment.

One of the main obstacles in cancer treatment is the resistance of cancer cells to anti-cancer therapy. miRNAs have been linked to the development of drug resistance in several cancers [38]. Recently, we demonstrated that miR-204 increases sensitivity of neuroblastoma cells to CDDP, in part, through the down-regulation of BCL2 [31]. MiR-497 has also been implicated in the development of multi-drug resistance in human gastric and lung cancer cell lines, at least in part, through targeting of anti-apoptotic BCL2 [21]. BCL2 has also been demonstrated as a direct target of miR-497 in neuroblastoma [22], although BCL2 knockdown alone only increases apoptosis in a cell line specific manner [31].

## Conclusions

Our study’s findings of the significant anti-proliferative effects of miR-497 further corroborate a tumor suppressive role for miR-497 in neuroblastoma, through the direct targeting of *WEE1*. Considering the significant increase in apoptosis due to WEE1 knock-down alone in MNA cell lines, or *WEE1* knockdown in combination with CDDP in both MNA and non-MNA cell lines, further investigation of *WEE1* as a potentially important therapeutic target in neuroblastoma is warranted.

## Materials and methods

### Primary neuroblastoma tumors

Primary neuroblastoma tumor samples (n=143) were obtained from the Children’s Oncology Group (COG), Philadelphia, USA (n=112) or from Our Lady’s Children’s Hospital, Dublin, Ireland (n=31) (Additional file 1: Table S1). Research was approved by the Research Ethics Committees of the Royal College of Surgeons and Our Lady’s Children’s Hospital, Dublin. Detailed miRNA expression profiling of this cohort of patients is described previously [9]. An independent data set of 88 primary neuroblastoma tumors was also used as part of the analysis for this study. This data is readily available using the web based R2 microarray analysis and visualization platform from the Academic Medical Center (AMC), Amsterdam (http://hgserver1.amc.nl/cgi-bin/r2/main.cgi).

### Cell culture and transfections

Neuroblastoma cell lines including Kelly and CHP-212 (*MYCN* amplified) and SK-N-AS (non-*MYCN* amplified) were purchased from the European Collection of Animal Cells. All lines were validated by short tandem repeat sequence genotyping and for presence of previously published genomic imbalances using array comparative genomic hybridisation (aCGH). Cell culture media was supplemented with 10% FBS and 1% Pen/Strep.

MiR-497 mimics and scrambled control oligonucleotides (Ambion, Life Technologies, Carlsbad, CA, USA) were transiently transfected in neuroblastoma cells at a final concentration of 30 nM by reverse transfection using siPORT™ *Neo*FX™ (Ambion). For siRNAs (siRNA negative control and siWEE1 final concentration 30nM (Ambion)), and in the co-transfection of luciferase reporter plasmids and miR mimics, cells were transiently transfected using Lipofectamine 2000 (Invitrogen).

### Cell viability and apoptosis assays

Viability of cells was measured by MTS-formazan reduction using CellTiter 96 Aqueous One Solution Cell Proliferation Assay (Promega, Madison, WI, USA) at 24 hr, 48 hr, 72 hr, and 96 hr post transfection. Absorbance was measured at 490 nm using a Synergy Multi-Mode Plate Reader (Boitek, Winooski, VT, USA). Apoptosis levels were demonstrated by Annexin-V staining and propidium iodide (PI) exclusion using the FITC Annexin-V Apoptosis Detection Kit I (BD Pharmingen, San Diego, CA, USA). Cells were acquired using a BD LSR II flow cytometer (Becton Dickinson, San Jose, CA, USA) and analysed using BD FACSDiva 4.0 Software. Caspase 3/7 activity was evaluated using the Caspase-Glo® 3/7 Assay (Promega) and luminescence recorded using a Synergy Multi-Mode Plate Reader (Boitek).

### Quantitative real-time RT-PCR

Total RNA was extracted from cell lines using miRNeasy Mini Kits (Qiagen, Valencia, CA, USA). Reverse transcription was performed using total RNA with primers specific for miR-497 or RNU48 control and TaqMan microRNA reverse transcription kit (Applied Biosystems Life Technologes, Carlsbad, California, USA). For gene expression analysis, reverse transcription was performed using High-Capacity reverse transcription kits (Applied Biosystems). Specific TaqMan assays (Applied Biosystems) for *WEE1* and miR-497 were employed for expression analysis on the 7900 HT Fast Realtime System (Applied Biosystems). MiRNA and gene expression was normalised using the endogenous controls RNU48 and 18S respectively and relative quantities determined by the delta CT method [39].

### Western blot analysis

Total protein was analysed by western blotting using primary antibodies anti-WEE1 (B11) (Santa Cruz Biotechnology, Santa Cruz, CA, U.S.A), followed by anti-mouse secondary antibody (Cell Signaling Technology, Beverly, MA USA) and anti-mouse alpha-tubulin loading control (Abcam,Cambridge, MA USA).

### Luciferase reporter assay

Direct targeting of the *WEE1* 3′UTRs was determined by cloning of the 3′UTR seed region and mutated seed regions into separate psiCHECK™-2 vectors (Eurofins MWG Operon, Anzingerstr Ebersberg Germany). Renilla and firefly luciferase activities were measured using the Dual-Luciferase® Reporter kit (Promega) and luminescence recorded on a Synergy Multi-Mode Plate Reader (Boitek).

### Cell cycle analysis

Cell cycle progression and proliferation was monitored using the Cell Cycle Assay Kit - Green Fluorometric at 48 hr post transfection (Abcam Cambridge, MA USA). Cells were acquired using a BD LSR II flow cytometer (Becton Dickinson, San Jose, CA, USA) and analysed using Weasel 3.1 Software

### Statistical analysis

All statistical analysis was performed using GraphPad prism 5 software (GraphPad Software, San Diego, CA, USA) or MedCalc Version 12.2.1.0 (MedCalc Software, Mariakerke, Belgium). A *P*-value of <0.05 was regarded as statistically significant (* p < 0.05; ** P < 0.01; *** P < 0.001).

## Abbreviations

INSS: International neuroblastoma staging system; miRNAs: MicroRNAs; UTR: Untranslated region; EFS: Event free survival; OS: Overall survival; MNA: *MYCN*-amplified; non-MNA: Non-*MYCN*-amplified; CDDP: Cisplatin; PI: Propidium iodide.

## Competing interests

The authors declare that they have no conflicting interests.

## Authors’ contributions

LC, JR, HH, IMB, MM, AK performed experiments; LC, JR and RLS made substantial contributions to the conception and design of experiments; LC, JR and RLS wrote the manuscript; all authors have made critical edits to the manuscript and have given final approval.

## Supplementary Material

Additional file 1: Table S1 Neuroblastoma Cohort Clinical Data. Click here for file

Additional file 2: Table S2 Multivariate (Cox proportional hazard regression) analysis of event free and overall survival in 143 neuroblastoma patients. Click here for file

Additional file 3: Figure S1 miR-497 mRNA expression following transfection with miR-497 mimics. Neuroblastoma *MYCN*-amplified cell lines Kelly (n=4), CHP-212 (n=4) and non-*MYCN*-amplified SK-N-AS (n=3) were transfected with miR-497 mimics/scrambled negative control (Neg Control) oligonucleotides. Upregulation of miR-497 mRNA expression levels compared to negative controls. Total RNA isolated 24 hrs post transfection. Click here for file

Additional file 4: Figure S4 Cell cycle analysis of MNA Kelly cells following miR-497 over-expression and siRNA mediated inhibition of WEE1. (A) Cell cycle analysis of MNA Kelly cells transfected with miR-497 mimics/scrambled negative controls (Neg Control) oligonucleotides. Mean percentage of cells in G_0_/G_1_ and G_2_/M phases of the cell cycle from three independent experiments at 48 hr post transfection. (B) Cell cycle analysis of MNA Kelly cells transfected with siWEE1/ siNegative control (siNEG Control). Mean percentage of cells in G_0_/G_1_ and G_2_/M phases of the cell cycle from three independent experiments at 48 hr post transfection. (C) Representative cell cycle plots for MNA Kelly following transfection with miR-497 mimics/scrambled negative control (Neg Control) oligonucleotides. (D) Representative cell cycle plots for MNA Kelly following transfection with siWEE1 /siNegative control (siNeg Control). Click here for file

Additional file 5: Figure S2 3′UTR sequences from WEE1 cloned into the luciferase reporter constructs. The miR-497 binding sites, or mutated sequence is underlined. Click here for file

Additional file 6: Figure S3 WEE1 mRNA and protein expression following siRNA mediated inhibition of WEE1**.** Neuroblastoma *MYCN*-amplified cell lines Kelly (n=3), CHP-212 (n=3) and non-*MYCN*-amplified SK-N-AS (n=3) were transfected with siWEE1/siNegative control (Neg Control) oligonucleotides (A) Downregulation of *WEE1* mRNA expression levels following siWEE1 compared to negative controls. Total RNA was isolated 24 hr post transfection. (B) Downregulation of WEE1 protein levels following siWEE1 compared to negative controls. Protein was isolated at 48 hrs post transfection. Click here for file
