# Supplementary material for: MicroRNA-497 increases apoptosis in MYCN amplified neuroblastoma cells by targeting the key cell cycle regulator WEE1
Source: Mol Cancer. 2013 Mar 26;12:23. doi: 10.1186/1476-4598-12-23 (PMC3626575; doi:10.1186/1476-4598-12-23)
Supplement: Additional file 1: Table S1 — Neuroblastoma Cohort Clinical Data. [file 1476-4598-12-23-S1.doc]

**Supplementary Table 1** Neuroblastoma Cohort Clinical Data (n=143)*.

| **UPN** | **AGE at Diagnosis** | **Chromosome 11q deletion** | **MYCN amplified** | **INSS** | **Grade** | **Relapse** |
| --- | --- | --- | --- | --- | --- | --- |
| NB1 | >=1 | Yes | no | Stage 1 | Low | no |
| NB2 | >=1 | Yes | no | Stage 1 | Low | no |
| NB3 | >=1 | normal | no | Stage 1 | Low | no |
| NB4 | <1 | normal | no | Stage 2 | Low | no |
| NB5 | <1 | normal | no | Stage 2 | Low | no |
| NB6 | >=1 | normal | no | Stage 2 | Low | no |
| NB7 | >=1 | normal | no | Stage 2 | Low | no |
| NB8 | >=1 | normal | no | Stage 2 | Low | no |
| NB9 | >=1 | normal | no | Stage 2 | Low | no |
| NB10 | >=1 | Yes | no | Stage 2 | Low | no |
| NB11 | <1 | normal | no | Stage 2 | Low | no |
| NB12 | >=1 | normal | no | Stage 2 | Low | no |
| NB13 | >=1 | normal | no | Stage 2 | Low | no |
| NB14 | <1 | normal | no | Stage 2 | Low | no |
| NB15 | <1 | normal | no | Stage 2 | Low | no |
| NB16 | <1 | normal | no | Stage 2 | Low | no |
| NB17 | >=1 | normal | no | Stage 2 | Low | no |
| NB18 | >=1 | normal | no | Stage 2 | Low | no |
| NB19 | >=1 | Yes | no | Stage 2 | Low | no |
| NB20 | >=1 | normal | no | Stage 2 | Low | no |
| NB21 | <1 | normal | no | Stage 2 | Low | no |
| NB22 | >=1 | normal | no | Stage 2 | Low | no |
| NB23 | <1 | normal | no | Stage 2 | Low | no |
| NB24 | <1 | normal | no | Stage 2 | Low | no |
| NB25 | >=1 | Yes | no | Stage 2 | Low | yes |
| NB26 | >=1 | Yes | no | Stage 2 | Low | no |
| NB27 | >=1 | normal | yes | Stage 2 | Low | no |
| NB28 | >=1 | Yes | no | Stage 2 | Low | no |
| NB29 | >=1 | normal | no | Stage 2 | Low | no |
| NB30 | >=1 | normal | no | Stage 2 | Low | no |
| NB31 | >=1 | Yes | no | Stage 2 | Low | no |
| NB32 | >=1 | Yes | no | Stage 2 | Low | no |
| NB33 | >=1 | normal | no | Stage 2 | Low | no |
| NB34 | >=1 | normal | yes | Stage 2 | Low | no |
| NB35 | >=1 | normal | yes | Stage 2 | Low | no |
| NB36 | <1 | normal | no | Stage 2 | Low | no |
| NB37 | >=1 | normal | no | Stage 2 | Low | no |
| NB38 | >=1 | normal | no | Stage 3 | Low | no |
| NB39 | >=1 | normal | yes | Stage 3 | Low | no |
| NB40 | >=1 | normal | no | Stage 3 | Low | no |
| NB41 | >=1 | normal | no | Stage 3 | Low | no |
| NB42 | >=1 | normal | no | Stage 3 | Low | no |
| NB43 | >=1 | normal | no | Stage 3 | Low | no |
| NB44 | >=1 | normal | yes | Stage 3 | Low | no |
| NB45 | >=1 | normal | no | Stage 3 | Low | no |
| NB46 | >=1 | normal | no | Stage 3 | Low | no |
| NB47 | >=1 | Yes | yes | Stage 3 | Low | yes |
| NB48 | >=1 | Yes | no | Stage 3 | Low | yes |
| NB49 | >=1 | normal | no | Stage 3 | Low | no |
| NB50 | >=1 | normal | no | Stage 3 | Low | yes |
| NB51 | >=1 | normal | yes | Stage 3 | Low | no |
| NB52 | >=1 | Yes | no | Stage 3 | Low | yes |
| NB53 | >=1 | normal | no | Stage 3 | Low | no |
| NB54 | >=1 | normal | no | Stage 3 | Low | no |
| NB55 | >=1 | normal | yes | Stage 3 | Low | no |
| NB56 | >=1 | normal | no | Stage 3 | Low | no |
| NB57 | >=1 | normal | no | Stage 3 | Low | no |
| NB58 | >=1 | normal | no | Stage 3 | Low | no |
| NB59 | >=1 | normal | yes | Stage 3 | Low | yes |
| NB60 | >=1 | Yes | no | Stage 3 | Low | no |
| NB61 | >=1 | normal | no | Stage 3 | Low | yes |
| NB62 | >=1 | normal | yes | Stage 3 | Low | no |
| NB63 | <1 | normal | no | Stage 3 | Low | no |
| NB64 | <1 | normal | no | Stage 3 | Low | no |
| NB65 | >=1 | normal | yes | Stage 3 | Low | yes |
| NB66 | >=1 | normal | yes | Stage 3 | Low | no |
| NB67 | >=1 | Yes | no | Stage 3 | Low | yes |
| NB68 | >=1 | normal | yes | Stage 3 | Low | no |
| NB69 | >=1 | normal | no | Stage 3 | Low | no |
| NB70 | >=1 | normal | no | Stage 4 | High | no |
| NB71 | >=1 | normal | no | Stage 4 | High | yes |
| NB72 | >=1 | normal | no | Stage 4 | High | yes |
| NB73 | >=1 | normal | yes | Stage 4 | High | yes |
| NB74 | >=1 | normal | yes | Stage 4 | High | yes |
| NB75 | >=1 | normal | no | Stage 4 | High | no |
| NB76 | >=1 | Yes | yes | Stage 4 | High | yes |
| NB77 | >=1 | Yes | no | Stage 4 | High | yes |
| NB78 | >=1 | Yes | no | Stage 4 | High | no |
| NB79 | >=1 | normal | no | Stage 4 | High | no |
| NB80 | >=1 | normal | yes | Stage 4 | High | no |
| NB81 | >=1 | Yes | yes | Stage 4 | High | yes |
| NB82 | >=1 | normal | no | Stage 4 | High | yes |
| NB83 | >=1 | Yes | no | Stage 4 | High | no |
| NB84 | >=1 | normal | no | Stage 4 | High | no |
| NB85 | >=1 | Yes | no | Stage 4 | High | no |
| NB86 | >=1 | Yes | no | Stage 4 | High | no |
| NB87 | >=1 | Yes | no | Stage 4 | High | no |
| NB88 | >=1 | normal | no | Stage 4 | High | no |
| NB89 | >=1 | normal | yes | Stage 4 | High | no |
| NB90 | >=1 | Yes | no | Stage 4 | High | no |
| NB91 | >=1 | normal | no | Stage 4 | High | yes |
| NB92 | >=1 | normal | no | Stage 4 | High | no |
| NB93 | >=1 | normal | no | Stage 4 | High | no |
| NB94 | >=1 | normal | no | Stage 4 | High | no |
| NB95 | >=1 | Yes | no | Stage 4 | High | no |
| NB86 | >=1 | Yes | no | Stage 4 | High | no |
| NB97 | >=1 | Yes | no | Stage 4 | High | no |
| NB98 | >=1 | normal | yes | Stage 4 | High | yes |
| NB99 | >=1 | normal | no | Stage 4 | High | no |
| NB100 | >=1 | Yes | no | Stage 4 | High | no |
| NB101 | >=1 | normal | yes | Stage 4 | High | yes |
| NB102 | >=1 | Yes | no | Stage 4 | High | yes |
| NB103 | >=1 | normal | yes | Stage 4 | High | no |
| NB104 | >=1 | normal | no | Stage 4 | High | no |
| NB105 | >=1 | normal | yes | Stage 4 | High | no |
| NB106 | >=1 | Yes | no | Stage 4 | High | yes |
| NB107 | >=1 | Yes | no | Stage 4 | High | no |
| NB108 | >=1 | normal | yes | Stage 4 | High | yes |
| NB109 | >=1 | normal | no | Stage 4 | High | no |
| NB110 | >=1 | normal | yes | Stage 4 | High | no |
| NB111 | >=1 | Yes | yes | Stage 4 | High | yes |
| NB112 | >=1 | normal | no | Stage 4 | High | no |
| NB113 | >=1 | Yes | no | Stage 4 | High | no |
| NB114 | >=1 | normal | yes | Stage 4 | High | yes |
| NB115 | <1 | Yes | no | Stage 4 | High | no |
| NB116 | >=1 | Yes | no | Stage 4 | High | no |
| NB117 | >=1 | Yes | no | Stage 4 | High | no |
| NB118 | >=1 | normal | yes | Stage 4 | High | yes |
| NB119 | >=1 | Yes | no | Stage 4 | High | no |
| NB120 | >=1 | Yes | no | Stage 4 | High | yes |
| NB121 | >=1 | Yes | no | Stage 4 | High | yes |
| NB122 | <1 | Yes | no | Stage 4 | High | yes |
| NB123 | <1 | Yes | no | Stage 4 | High | yes |
| NB124 | >=1 | normal | yes | Stage 4 | High | yes |
| NB125 | >=1 | Yes | no | Stage 4 | High | yes |
| NB126 | >=1 | Yes | no | Stage 4 | High | yes |
| NB127 | >=1 | Yes | no | Stage 4 | High | yes |
| NB128 | <1 | normal | yes | Stage 4 | High | no |
| NB129 | >=1 | normal | yes | Stage 4 | High | yes |
| NB130 | >=1 | Yes | no | Stage 4 | High | yes |
| NB131 | >=1 | normal | yes | Stage 4 | High | no |
| NB132 | >=1 | normal | yes | Stage 4 | High | yes |
| NB133 | >=1 | Yes | no | Stage 4 | High | no |
| NB134 | <1 | normal | no | Stage 4 | High | no |
| NB135 | >=1 | Yes | no | Stage 4 | High | no |
| NB136 | <1 | normal | no | Stage 4 | High | no |
| NB137 | <1 | normal | no | Stage 4 | High | no |
| NB138 | >=1 | Yes | no | Stage 4 | High | no |
| NB139 | >=1 | normal | no | Stage 4 | High | no |
| NB140 | >=1 | Yes | no | Stage 4 | High | yes |
| NB141 | >=1 | Yes | no | Stage 4 | High | yes |
| NB142 | >=1 | Yes | no | Stage 4s | Low | no |
| NB143 | <1 | normal | no | Stage 4s | Low | no |

*All samples for analysis were obtained at time of diagnosis
