# Supplementary material for: MicroRNA-497 increases apoptosis in MYCN amplified neuroblastoma cells by targeting the key cell cycle regulator WEE1
Source: Mol Cancer. 2013 Mar 26;12:23. doi: 10.1186/1476-4598-12-23 (PMC3626575; doi:10.1186/1476-4598-12-23)
Supplement: Additional file 2: Table S2 — Multivariate (Cox proportional hazard regression) analysis of event free and overall survival in 143 neuroblastoma patients. [file 1476-4598-12-23-S2.doc]

**Supplementary Table 2.** Multivariate (Cox proportional hazard regression) analysis of event free and overall survival in 143 neuroblastoma patients

|  | |  | **EFS** | | | | | | **OS** | | | | | |
| --- | --- | --- | --- | --- | --- | --- | --- | --- | --- | --- | --- | --- | --- | --- |
| **Variable*** | | **n** | **Entered** | | | **Stepwise**** | | | **Entered** | | | **Stepwise**** | | |
|  | |  | **HR** | **95% CI** | **EFS *P*** | **HR** | **95% CI** | **EFS *P*** | **HR**** | **95% CI** | **EFS *P*** | **HR** | **95% CI** | **EFS *P*** |
| **INSS** | Stage 4  Stage 1,2,3,4S | 72  71 | 4.86  1 | 2.03-11.63 | 0.0004 | 6.46  1 | 3.0-13.8 | <0.0001 | 12.44  1 | 3.56-43.36 | 0.0001 | 12.9  1 | 4.37-38.0 | <0.0001 |
|  |  |  |  |  |  |  |  |  |  |  |  |  |  |  |
| **Age** | ≥1 year  <1 year | 123  20 | 2.72  1 | 0.63-11.6 | 0.1788 | - | - | - | 6.94  1.. | 0.91-52.9 | 0.0629 | - | - | - |
|  |  |  |  |  |  |  |  |  |  |  |  |  |  |  |
| **MYCN** | Amplified  Non Amplified | 33  110 | 2.65  1 | 1.28-5.49 | 0.0089 | 2.55  1 | 1.3-4.8 | 0.0043 | 2.19  1 | 0.91-5.29 | 0.0810 | 3.15  1 | 1.47-6.73 | 0.0031 |
|  |  |  |  |  |  |  |  |  |  |  |  |  |  |  |
| **11q** | Deletion  No Deletion | 50  93 | 1.36  1 | 0.64-2.91 | 0.4267 | - | - | - | 0.68  1 | 0.27-1.68 | 0.4115 | - | - | - |
|  |  |  |  |  |  |  |  |  |  |  |  |  |  |  |
| **miR-497** | Low Expression  High Expression | 36  106 | 1.39  1 | 0.69-2.83 | 0.358 | - | - | - | 1.71  1 | 0.70-4.07 | 0.1822 | - | - | - |
|  |  |  |  |  |  |  |  |  |  |  |  |  |  |  |
|  | < Median Expression | 72 | 0.86 | 0.44-1.68 | 0.6736 | - | - | - | 0.97 | 0.44-2.15 | 0.9521 | - | - | - |
|  | > Median Expression | 71 | 1 |  |  |  |  |  | 1 |  |  |  |  |  |

*Variables entered in Cox proportional hazard model; miR-497 expression (low expression < first quartile , high expression > first quartile, or >/< median expression), INSS (Stage 4, Stages 1,2,3,4S) Age (≥1 year, <1 year), MYCN amplified (yes, no), Chromosome 11q deleted (yes, no). **The variables Age, 11q status, and miR-497 were found not to significantly contribute to the prediction of event free or overall survival time and were excluded in the stepwise Cox multivariate model. EFS= Event free survival, OS= overall survival, HR= Hazard Ratio, 95% CI= 95% Confidence Interval.
