# Supplementary figures and images for: MicroRNA-497 increases apoptosis in MYCN amplified neuroblastoma cells by targeting the key cell cycle regulator WEE1
Source: Mol Cancer. 2013 Mar 26;12:23. doi: 10.1186/1476-4598-12-23 (PMC3626575; doi:10.1186/1476-4598-12-23)

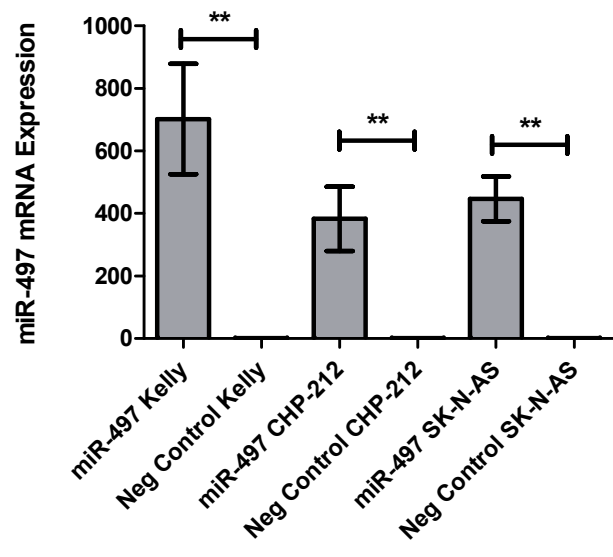

Supplement: Additional file 3: Figure S1 — miR-497 mRNA expression following transfection with miR-497 mimics. Neuroblastoma MYCN-amplified cell lines Kelly (n=4), CHP-212 (n=4) and non-MYCN-amplified SK-N-AS (n=3) were transfected with miR-497 mimics/scrambled negative control (Neg Control) oligonucleotides. Upregulation of miR-497 mRNA expression levels compared to negative controls. Total RNA isolated 24 hrs post transfection. [file 1476-4598-12-23-S3.pdf]

**A**

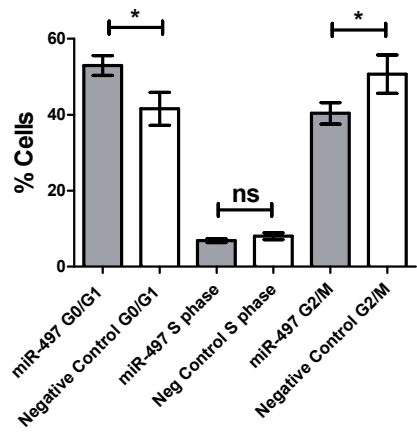

**B**

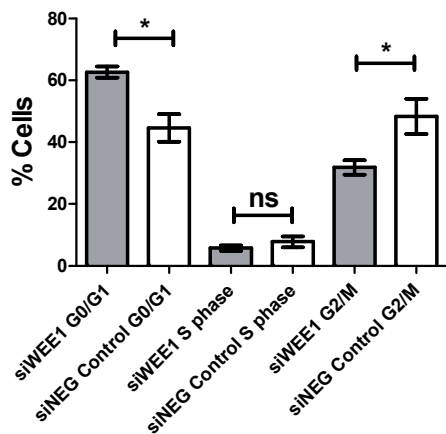

**C**

**miR-497 Kelly**

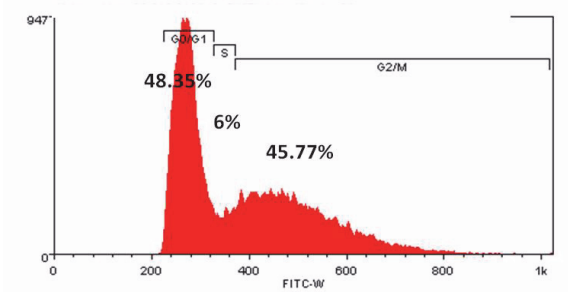

**Neg Control Kelly**

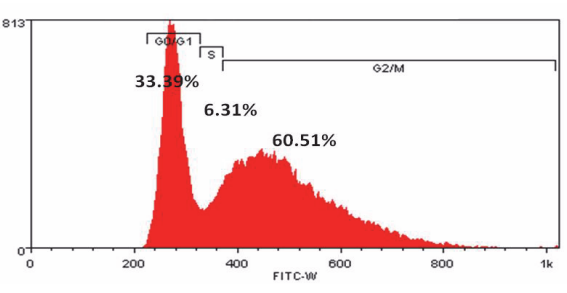

**D**

**siWEE1 Kelly**

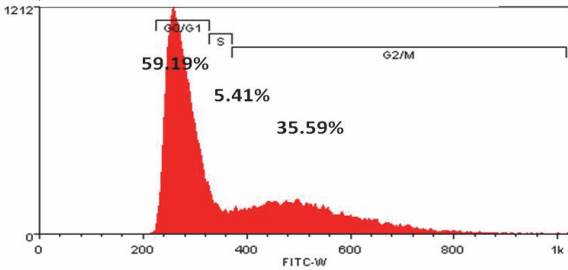

**siNEG Control Kelly**

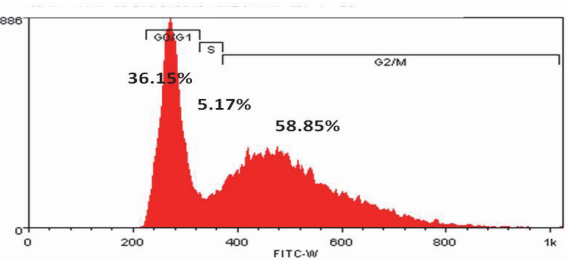

Supplement: Additional file 4: Figure S4 — Cell cycle analysis of MNA Kelly cells following miR-497 over-expression and siRNA mediated inhibition of WEE1. (A) Cell cycle analysis of MNA Kelly cells transfected with miR-497 mimics/scrambled negative controls (Neg Control) oligonucleotides. Mean percentage of cells in G0/G1 and G2/M phases of the cell cycle from three independent experiments at 48 hr post transfection. (B) Cell cycle analysis of MNA Kelly cells transfected with siWEE1/ siNegative control (siNEG Control). Mean percentage of cells in G0/G1 and G2/M phases of the cell cycle from three independent experiments at 48 hr post transfection. (C) Representative cell cycle plots for MNA Kelly following transfection with miR-497 mimics/scrambled negative control (Neg Control) oligonucleotides. (D) Representative cell cycle plots for MNA Kelly following transfection with siWEE1 /siNegative control (siNeg Control). [file 1476-4598-12-23-S4.pdf]

**A**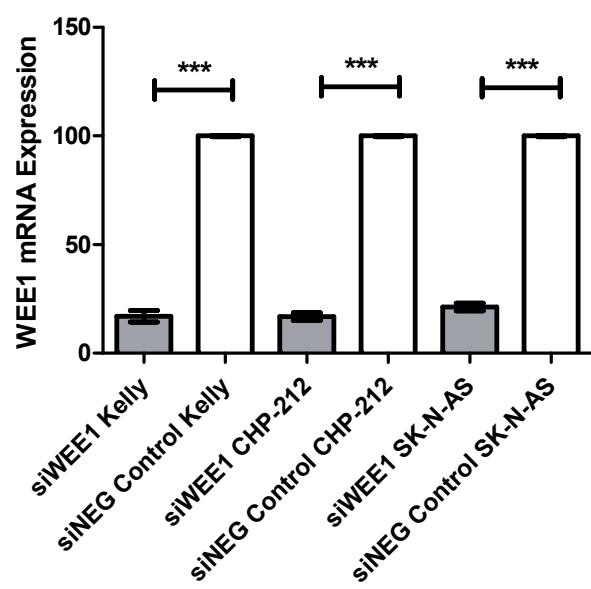**B**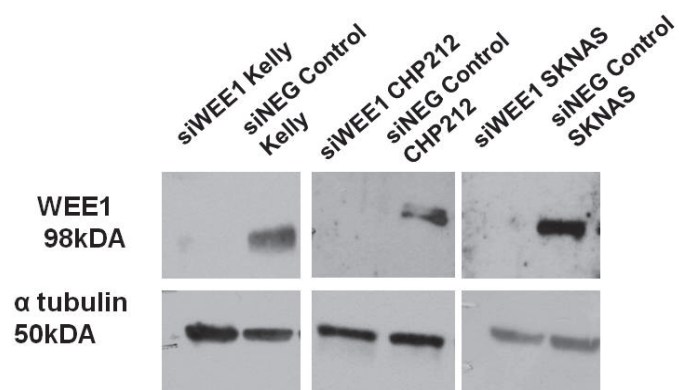

Supplement: Additional file 6: Figure S3 — WEE1 mRNA and protein expression following siRNA mediated inhibition of WEE1. Neuroblastoma MYCN-amplified cell lines Kelly (n=3), CHP-212 (n=3) and non-MYCN-amplified SK-N-AS (n=3) were transfected with siWEE1/siNegative control (Neg Control) oligonucleotides (A) Downregulation of WEE1 mRNA expression levels following siWEE1 compared to negative controls. Total RNA was isolated 24 hr post transfection. (B) Downregulation of WEE1 protein levels following siWEE1 compared to negative controls. Protein was isolated at 48 hrs post transfection. [file 1476-4598-12-23-S6.pdf]
