# Supplementary material for: MicroRNA-497 increases apoptosis in MYCN amplified neuroblastoma cells by targeting the key cell cycle regulator WEE1
Source: Mol Cancer. 2013 Mar 26;12:23. doi: 10.1186/1476-4598-12-23 (PMC3626575; doi:10.1186/1476-4598-12-23)
Supplement: Additional file 5: Figure S2 — 3′UTR sequences from WEE1 cloned into the luciferase reporter constructs. The miR-497 binding sites, or mutated sequence is underlined. [file 1476-4598-12-23-S5.pdf]

Supplementary Figure S2: 3'UTR sequences from *WEE1* cloned into the luciferase reporter constructs.

WEE1 luciferase plasmid insert with wild-type miR-497 binding sites: (WT WEE1) (NM_001143976)

5'
atttgatgattgctatgtcaggctttcatctaatcttaccagtctgtcttctgtaggatgtgtcactgttggat
gttacaccagcctttccagggttaaccactgtggtggtgtgctgcttatagtttgctgttgcattgtaataaaag
gtgtctttccctgtagtgacctgtaaaaagtactcaagggctttattacagacataccctccctttgaaaaggga
catgctaaaagactcattactactcagccttcaatgtacctgtgtgtccatcttatatttctttttttttttaatt
gtgaattagacttgtatatcccactgggagcactttgtaggcattgcatgaaccatgggatgatgattctgtgga
ggtattgccttgtgaatttgctgctattttagttttgtctttgctgtaaacttgtagcattaaacaatcattgtt
3'

WEE1 luciferase plasmid insert with first miR-497 binding site mutated: (Mut1 WEE1)

5'
atttgatgattgctatgtcaggctttcatctaatcttaccagtctgtcttctgtaggatgtgtcactgttggat
gttacaccagcctttccagggttaaccactgtggtggtgGTAtTTCtatagtttgctgttgcattgtaataaaag
gtgtctttccctgtagtgacctgtaaaaagtactcaagggctttattacagacataccctccctttgaaaaggga
catgctaaaagactcattactactcagccttcaatgtacctgtgtgtccatcttatatttctttttttttttaatt
gtgaattagacttgtatatcccactgggagcactttgtaggcattgcatgaaccatgggatgatgattctgtgga
ggtattgccttgtgaatttgctgctattttagttttgtctttgctgtaaacttgtagcattaaacaatcattgtt
3'

WEE1 luciferase plasmid insert with both miR-497 binding sites mutated: (Double Mut WEE1)

5'
atttgatgattgctatgtcaggctttcatctaatcttaccagtctgtcttctgtaggatgtgtcactgttggat
gttacaccagcctttccagggttaaccactgtggtggtgGTAtTTCtatagtttgctgttgcattgtaataaaag
gtgtctttccctgtagtgacctgtaaaaagtactcaagggctttattacagacataccctccctttgaaaaggga
catgctaaaagactcattactactcagccttcaatgtacctgtgtgtccatcttatatttctttttttttttaatt
gtgaattagacttgtatatcccactgggagcactttgtaggcattgcatgaaccatgggatgatgattctgtgga
ggtattgccttgtgaattGAAtATCattttagttttgtctttgctgtaaacttgtagcattaaacaatcattgtt
3'

WEE1 luciferase plasmid insert with second miR-497 binding site mutated: (Mut2 WEE1)

5'
atttgatgattgctatgtcaggctttcatctaatcttaccagtctgtcttctgtaggatgtgtcactgttggat
gttacaccagcctttccagggttaaccactgtggtggtgtgctgcttatagtttgctgttgcattgtaataaaag
gtgtctttccctgtagtgacctgtaaaaagtactcaagggctttattacagacataccctccctttgaaaaggga
catgctaaaagactcattactactcagccttcaatgtacctgtgtgtccatcttatatttctttttttttttaatt
gtgaattagacttgtatatcccactgggagcactttgtaggcattgcatgaaccatgggatgatgattctgtgga
ggtattgccttgtgaattGAAtATCattttagttttgtctttgctgtaaacttgtagcattaaacaatcattgtt
3'
